# The National Medical Union

**Published:** 1916-05-27

**Authors:** 


### The National Medical Union.

(*Communicated.*)

At a special general meeting of the National Medical Union, held at its offices, 346 Strand, on May 16, 1916, under the presidency of Mr. V. T. Greenyer, F.R.C.S., Chairman of Council, the following resolutions were passed unanimously:

(A) That the National Medical Union expresses its strong disapproval of the action of the Local Government Board in attempting to effect any reduction in the fees paid to the medical profession for the notification of infectious diseases, being convinced that at the present time complete harmony is necessary between the Public Health Service and the civil profession in order to control the spread of infectious diseases, and, in view of the fact that the profession is at present giving other certificates in relation to infectious diseases gratuitously—viz., certificates for removal of patients to hospital, for disinfection of premises, and for return to school—it reserves to itself the right to decline to furnish these extra certificates, unless adequately remunerated by the authority demanding them.

(B) That the National Medical Union sincerely hopes that the Government will take steps to reduce the duty on motor-car licences payable by the medical profession to that of 1915, on the grounds that the car is absolutely essential to medical men (1) to enable them to see the increased number of patients caused by undertaking both the private and hospital work of colleagues on active service, (2) to carry out the duties entailed in country practices extending over a large area, and (3) in view of the primary importance of prompt attendance on urgent cases.

(C) That the National Medical Union trusts that the Government will arrange to supply the medical profession with petrol at as low a cost as possible consistent with circumstances. They note with regret that the question of petrol supply has been placed in the hands of the National Health Insurance Commission. In these circumstances a large number of medical men have declined to respond to the notices issued by that body.

